# Excision of a Giant Cell Tumor With Bone Grafting and Bone Cementing of the Proximal Humerus: A Case Report

**DOI:** 10.7759/cureus.59492

**Published:** 2024-05-01

**Authors:** Milind R Gharpinde, Gajanan Pisulkar, Shounak Taywade, Abhiram A Awasthi, Anmol Suneja

**Affiliations:** 1 Orthopaedics, Jawaharlal Nehru Medical College, Datta Meghe Institute of Higher Education and Research, Wardha, IND

**Keywords:** post-operative care, surgical excision, bone cementing, bone grafting, proximal humerus, giant cell tumor

## Abstract

Giant cell tumors (GCTs) of the bone are uncommon neoplasms that predominantly affect the metaphysis of long bones, with proximal humerus involvement being less frequent. We present the case of a 58-year-old male who presented with a two-month history of progressive right shoulder pain and difficulty in raising his arm. Clinical examination revealed a palpable swelling on the lateral aspect of the right arm. Radiological investigations, including X-ray and magnetic resonance imaging (MRI), confirmed the presence of a primary osseous neoplasm involving the proximal humerus, suggestive of a GCT. The patient underwent surgical excision of the tumor with bone grafting and bone cementing of the proximal humerus. Post-operative care included prescribed medications and physiotherapy. This case highlights the successful management of GCTs of the proximal humerus through a multidisciplinary approach, emphasizing the importance of meticulous surgical technique, appropriate reconstruction, and comprehensive post-operative care for optimal patient outcomes.

## Introduction

Giant cell tumors (GCTs) are locally aggressive, benign bone tumors that primarily affect the metaphysis of long bones, with the distal femur, proximal tibia, and distal radius being the most commonly involved sites [1]. However, GCTs occurring in the proximal humerus represent a less frequent but clinically significant entity, accounting for approximately 3-5% of all GCT cases [2]. These tumors typically arise in skeletally mature individuals between the ages of 20 and 50 years, with a slight predilection for females [3]. Histologically, GCTs are characterized by a proliferation of multinucleated giant cells within a background of mononuclear stromal cells, which may exhibit varying degrees of mitotic and osteoclastic activity [4]. Despite their benign nature, GCTs tend to local recurrence, with rates ranging from 10% to 50%, depending on factors such as tumor size, location, and adequacy of surgical resection [5].

The clinical presentation of GCTs typically includes localized pain, swelling, and limited range of motion at the affected site. In proximal humerus cases, patients may experience difficulty in shoulder movement and lifting objects overhead. Radiographically, GCTs often present as lytic lesions with well-defined margins and eccentric cortical thinning, occasionally exhibiting cortical breakthrough and soft tissue extension [6]. The management of GCTs of the proximal humerus poses unique challenges due to the anatomical complexity of the shoulder region and the risk of compromising adjacent neurovascular structures. Surgical excision with wide or intralesional margins remains the cornerstone of treatment, aiming to achieve complete tumor removal while preserving joint function and minimizing the risk of recurrence [7]. Various surgical techniques have been employed for the treatment of GCTs in the proximal humerus, including curettage with or without adjuvant therapies (such as phenol, liquid nitrogen, or polymethylmethacrylate), en bloc resection with reconstruction, and minimally invasive approaches [8]. The choice of surgical approach depends on several factors, including tumor size, location, presence of pathological fracture, and surgeon expertise.

In recent years, advancements in imaging modalities, such as magnetic resonance imaging (MRI) and computed tomography (CT), have improved pre-operative planning and intra-operative navigation, facilitating more precise tumor localization and resection [9]. Developing novel adjuvant therapies and targeted molecular agents also holds promise for improving outcomes and reducing recurrence rates in patients with GCTs. In this context, we present the case report of a 58-year-old male patient with a GCT of the proximal humerus who underwent surgical excision with bone grafting and bone cementing, highlighting the clinical presentation, operative technique, and post-operative management of this rare but clinically significant condition.

## Case presentation

**Figure 1:**
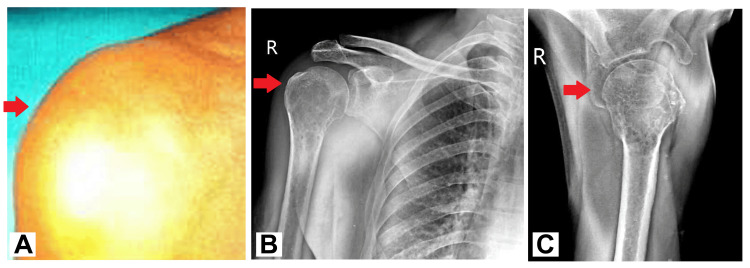
(A) Clinical photographs. (B-C) X-ray of the right shoulder with arm AP and lateral view showing the homogenous radio-opaque area in the proximal end of the humerus AP: anteroposterior

The case involves a 58-year-old male patient who presented with a chief complaint of persistent right shoulder pain, which had been troubling him for the past two months. The pain was gradually progressive in nature, and he experienced increasing difficulty in raising his arm. The patient reported no significant past medical history or trauma to the shoulder region. However, over the past month, the pain had intensified to the extent that it significantly hindered his daily activities. Upon physical examination, a notable finding was a widespread swelling on the lateral aspect of the right arm, measuring approximately 6 × 8 cm (Figure 1A). The swelling was accompanied by localized discomfort over the bulge. Given the persistence and worsening of symptoms, further investigation was warranted. X-ray imaging of the right shoulder with arm anteroposterior (AP) and lateral views revealed a homogenous radio-opaque area in the proximal end of the humerus, resembling a soap bubble appearance (Figure 1B, 1C). Subsequent MRI of the right shoulder confirmed the presence of a primary osseous neoplasm involving the proximal humerus, extending towards the subarticular region of the humeral head. Given its characteristic appearance and location, the radiological findings raised suspicion of a GCT.

**Figure 2:**
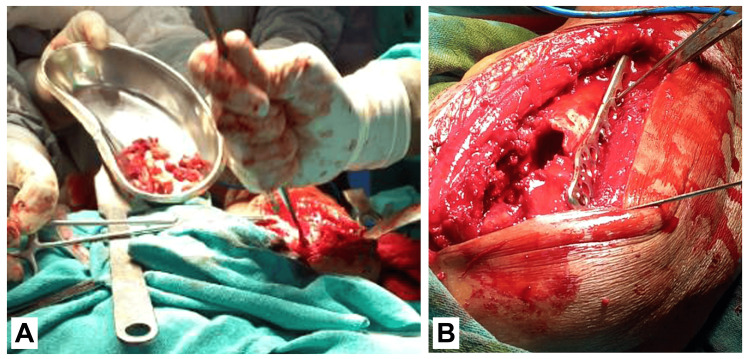
(A) Intra-operative figure of the procedure. (B) Intra-images of bone grafting and PHILOS plating PHILOS: proximal humeral internal locking system

Following the diagnosis, the patient underwent an operative procedure for the excision of the GCT with bone grafting and bone cementing of the proximal humerus. Under general anesthesia, he was positioned supine on the operating table, and the right upper limb and right hip were meticulously prepared and draped under aseptic conditions. A 5 cm incision was made at the right iliac crest to obtain bone graft material, followed by a 10 cm incision over the proximal humerus. Soft tissue dissection was performed, and a cortical window was created to access the tumor. Multiple drilled holes were made in the anterior cortex of the humerus head, facilitating the removal of the hemorrhagic tumor mass. After thorough debridement and tumor excision, a proximal humeral internal locking system (PHILOS) plate was placed to provide stability, and the surgical site was filled with bone graft and bone cement (Figure 2A, 2B). Closure was performed in layers, and the patient was transferred to the recovery room for post-operative observation.

**Figure 3:**
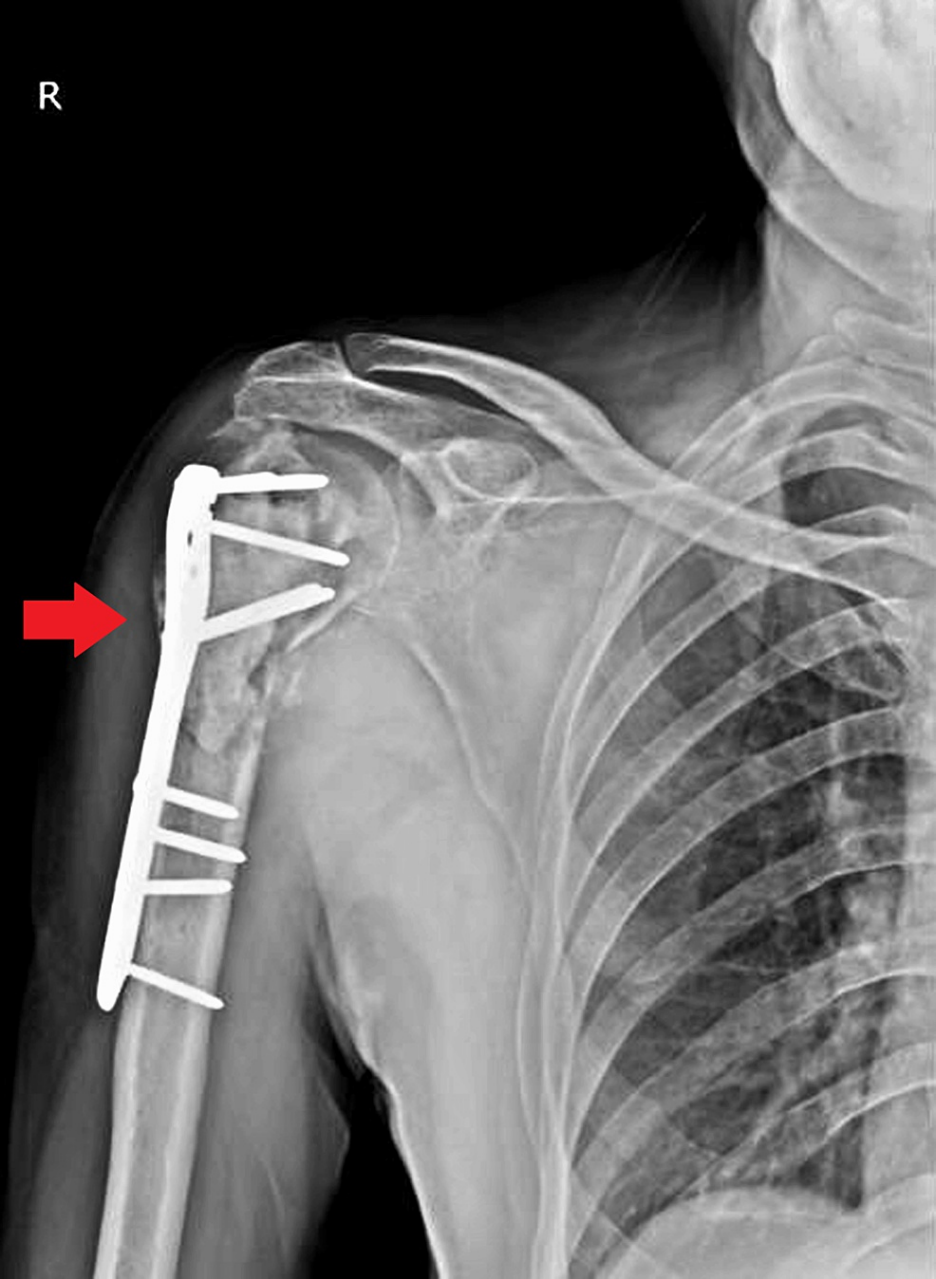
Post-operative X-ray of the right shoulder having a bone graft, a bone cement, and a PHILOS plate PHILOS: proximal humeral internal locking system

In the post-operative period, the patient was instructed to continue prescribed oral medications and initiate regular physiotherapy sessions focusing on shoulder and wrist range of motion exercises. Figure 3 shows the post-operative X-ray of the right shoulder, which has a bone graft, a bone cement, and a PHILOS plate. Emphasis was placed on keeping the right upper limb elevated and avoiding heavy lifting with the right hand to prevent undue strain on the surgical site.

## Discussion

GCTs of the bone are relatively rare but locally aggressive neoplasms that most commonly affect the metaphysis of long bones, particularly around the knee joint [10]. However, involvement of the proximal humerus, as seen in this case, is less frequent but poses significant challenges due to the anatomical complexity and proximity to vital structures such as the brachial plexus and axillary vessels [10]. Surgical excision with adequate margins remains the cornerstone of treatment for GCTs to prevent recurrence and preserve limb function [11]. In this case, the operative approach involved the excision of the tumor with bone grafting and bone cementing of the proximal humerus. This technique aims to achieve complete tumor removal while providing structural support and stability to the affected bone segment [12].

Using bone grafts from the iliac crest and bone cementing techniques have been widely adopted in orthopedic oncology to reconstruct skeletal defects following tumor resection [13]. Incorporating bone grafts enhances osseous healing and promotes bone regeneration, while bone cement provides immediate structural support and helps fill any voids left after tumor removal [14]. The choice of implant for fixation, in this case, was a PHILOS plate, a commonly used fixation device in proximal humeral fractures. Its application in this context provides stability to the humeral head, particularly in cases with a fracture risk following tumor excision [15].

Post-operative care ensures optimal outcomes and includes a combination of prescribed medications and physiotherapy. Regular physiotherapy sessions focusing on motion exercises are essential for restoring shoulder function and preventing stiffness [16]. Long-term follow-up is imperative in managing GCTs to monitor for recurrence and assess functional outcomes. Despite the favorable outcome in this case, the risk of recurrence remains a concern, necessitating regular surveillance with imaging studies such as X-rays and MRI [17].

## Conclusions

The successful excision of the GCT with bone grafting and bone cementing of the proximal humerus, in this case, underscores the importance of a comprehensive approach to managing such neoplasms. Through meticulous surgical techniques, including tumor removal with adequate margins and reconstruction using bone grafts and cement, optimal outcomes can be achieved, ensuring structural stability and preserving limb function. Furthermore, implementing post-operative care, including prescribed medications and physiotherapy, is essential for facilitating rehabilitation and preventing complications. Although the risk of recurrence remains a concern, long-term follow-up and surveillance are imperative for timely detection and intervention. Overall, this case highlights the efficacy of multidisciplinary collaboration and emphasizes the significance of individualized treatment strategies in optimizing patient outcomes and quality of life.
